# Optical Coherence Tomography-Assisted Diagnosis and Optimal Treatment of Three Patients With Woven Coronary Artery

**DOI:** 10.7759/cureus.80648

**Published:** 2025-03-16

**Authors:** Yunfei Guo, Ping Han, Xuefeng Geng, Hongxu Zhu, Fuchun Zhang

**Affiliations:** 1 Department of Cardiology, Beijing Haidian Hospital, Beijing, CHN; 2 Department of Cardiology and Institute of Vascular Medicine, Peking University Third Hospital, Beijing, CHN

**Keywords:** coronary artery angiography, oct (optical coherence tomography), recanalized thrombus, spontaneous coronary dissection, woven coronary artery

## Abstract

Woven coronary artery (WCA) is an infrequent coronary artery anomaly characterized by the segmentation of one or more coronary artery portions into multiple small channels by intertwining small blood vessels, which reconverge into a normal lumen in the distal segment. Coronary angiography is not sufficiently accurate for the definitive diagnosis of "braid-like lesions." Optical coherence tomography (OCT) examination provides a clearer understanding of the coronary artery conditions.

We reported on three cases that exhibited manifestations similar to Woven syndrome during coronary angiography. After OCT examination, in one of the cases, there was a presence of thrombus and cavities of varying sizes in the coronary artery, with intercommunication between the cavities and no apparent tunica media, suggesting organized thrombus. The patient underwent stent implantation treatment. The other two cases were confirmed to have relatively intact three-layer vascular structures within each channel and a significant absence of important coronary atherosclerotic disease upon OCT examination, leading to a diagnosis of Woven syndrome. They were then treated with optimal medical therapy.

In the context of coronary angiography, woven coronary artery (WCA) presents as "honeycomb," "braid-like," and "figure 8" configurations, often mimicking spontaneous coronary dissection or recanalized thrombus. Optical coherence tomography (OCT) emerges as an indispensable diagnostic adjunct, providing confirmatory evidence and mitigating the risk of procedural complications associated with misdiagnosis and facilitating the selection of therapeutic strategies. This report discusses the differential diagnosis, diagnostic challenges, and treatment considerations for WCA.

## Introduction

Woven coronary artery (WCA) is a rare clinical coronary artery malformation, wherein segments of the coronary artery are partitioned into several smaller channels by a network of interwoven small blood vessels. These channels subsequently merge to form a normal lumen in the distal segment of the coronary artery. The differential diagnosis for WCA includes spontaneous coronary dissection (SCAD), recanalized thrombus, and bridging collateral vessels. SCAD is a condition where a tear or separation occurs within the layers of the coronary artery wall, leading to the formation of a hematoma (a collection of blood) within the arterial wall. While most coronary artery anomalies are diagnosed using coronary angiography (CAG), distinguishing between woven-like changes and recanalized thrombus via CAG can be challenging. Optical coherence tomography (OCT) has been shown to enhance diagnostic accuracy in such cases [1,2]. We reported on three cases that exhibited manifestations similar to Woven syndrome during coronary angiography. Following the OCT examination, one patient was confirmed to have an organized thrombus and underwent interventional treatment. The other two cases were diagnosed with Woven syndrome and were treated with optimal medical treatment (OMT). The following case reports describe three patients who all exhibited woven-like changes on coronary angiography, but the OCT findings and therapeutic strategies were different.

## Case presentation

Case 1

**Figure 1:**
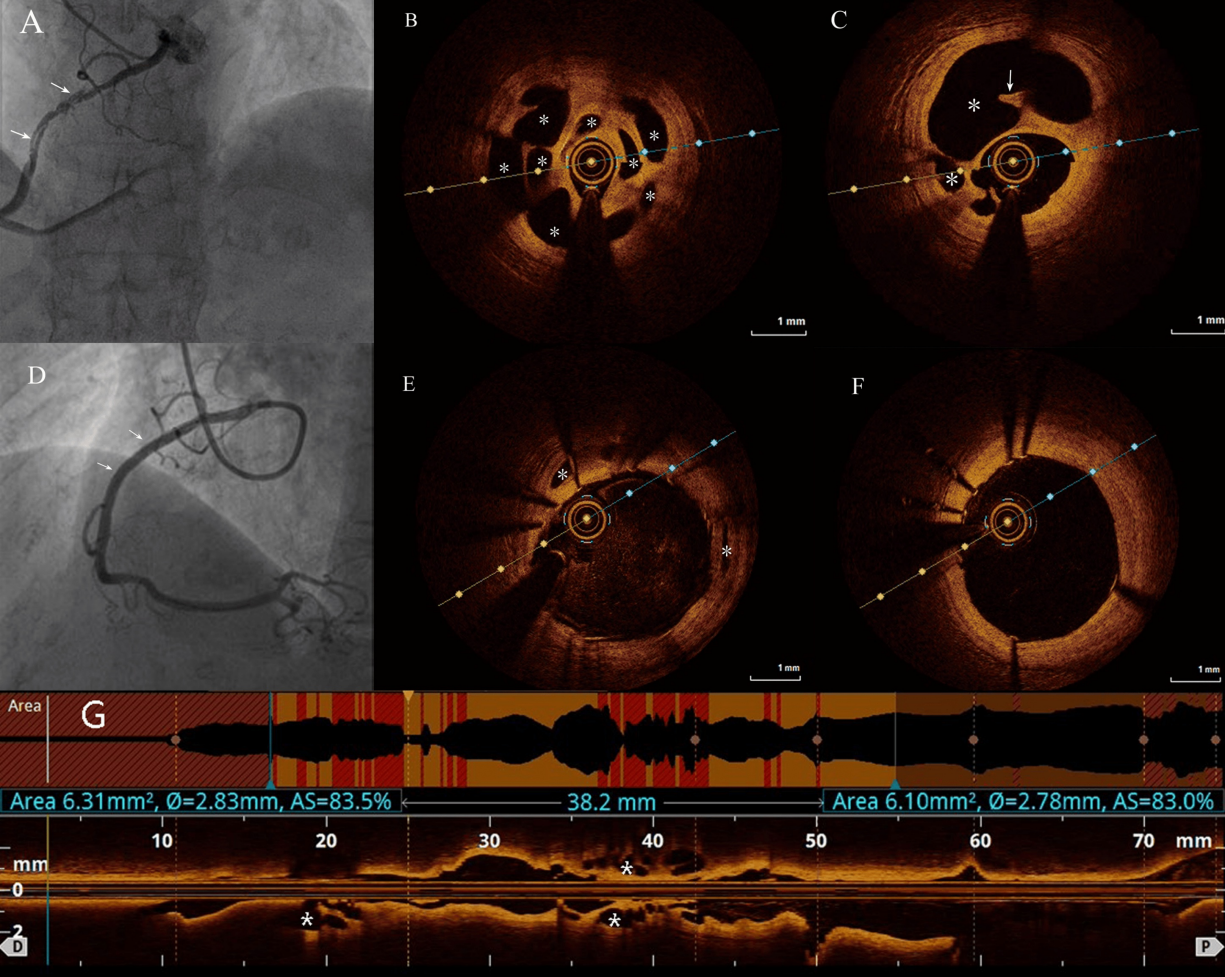
Angiography and optical coherence tomography (OCT) images of the RCA A: coronary angiogram before stent implantation (arrows indicate the small, twisting channels); B, C, G: OCT revealed white thrombus (arrow) and cavities of varying sizes in the proximal segment of the RCA (asterisks), with intercommunication between the cavities and no apparent tunica media; D: images after intervention therapy, PCI was performed with a 3.0 mm×38 mm DES implanted in the proximal of the RCA (arrow), and there were no interventional complications; E: OCT re-examination found that the intertwined small blood vessels around the main lumen in the proximal RCA artery (shown as cavities before) were almost occluded (asterisk); F: OCT confirmed well-adhered and expanded coronary stent. CAG: coronary angiography; OCT: optical coherence tomography; PCI: percutaneous coronary intervention; RCA: right coronary artery; DES: drug-eluting stent

A 66-year-old female was admitted to the cardiology department of Beijing Haidian Hospital because of III/IV Canadian cardiovascular society (CCS) stable angina despite optimal medical treatment. The patient had a four-year history of hypertension and hyperlipidemia but denied a history of diabetes and smoking. The exercise test showed ST segment depression in leads II, III, and augmented voltage foot (AVF) lasting for more than two minutes, and coronary angiography was performed. The right coronary artery (RCA) showed an intraluminal filling defect and a vague tortuous appearance in the proximal segment with thrombolysis in myocardial infarction (TIMI) 3 flow (Figure 1A). However, no significant abnormalities were observed in the left anterior descending artery (LAD) and the left circumflex artery (LCX). In order to better understand the anatomical characteristics of coronary arteries, an OCT examination of the RCA was performed. The result of OCT demonstrated that the lumina were clearly inside the tunica media. Both the longitudinal and cross-sectional views reveal the presence of thrombus and cavities of varying sizes within the coronary artery, with intercommunication between the cavities and no apparent tunica media, suggesting organized thrombus (Figures 1B, 1C, 1G). Considering the symptoms of the patient and the results of the exercise test, the RCA was considered to be the coronary artery related to the angina. A 3.0 mm × 38 mm drug-eluting stent was implanted in the proximal and middle segments of the RCA, and OCT showed a well-deployed stent with diminished additional channels (Figures 1D-1F). During the follow-up period of six months, the patient remained asymptomatic and had no adverse events.

Case 2

**Figure 2:**
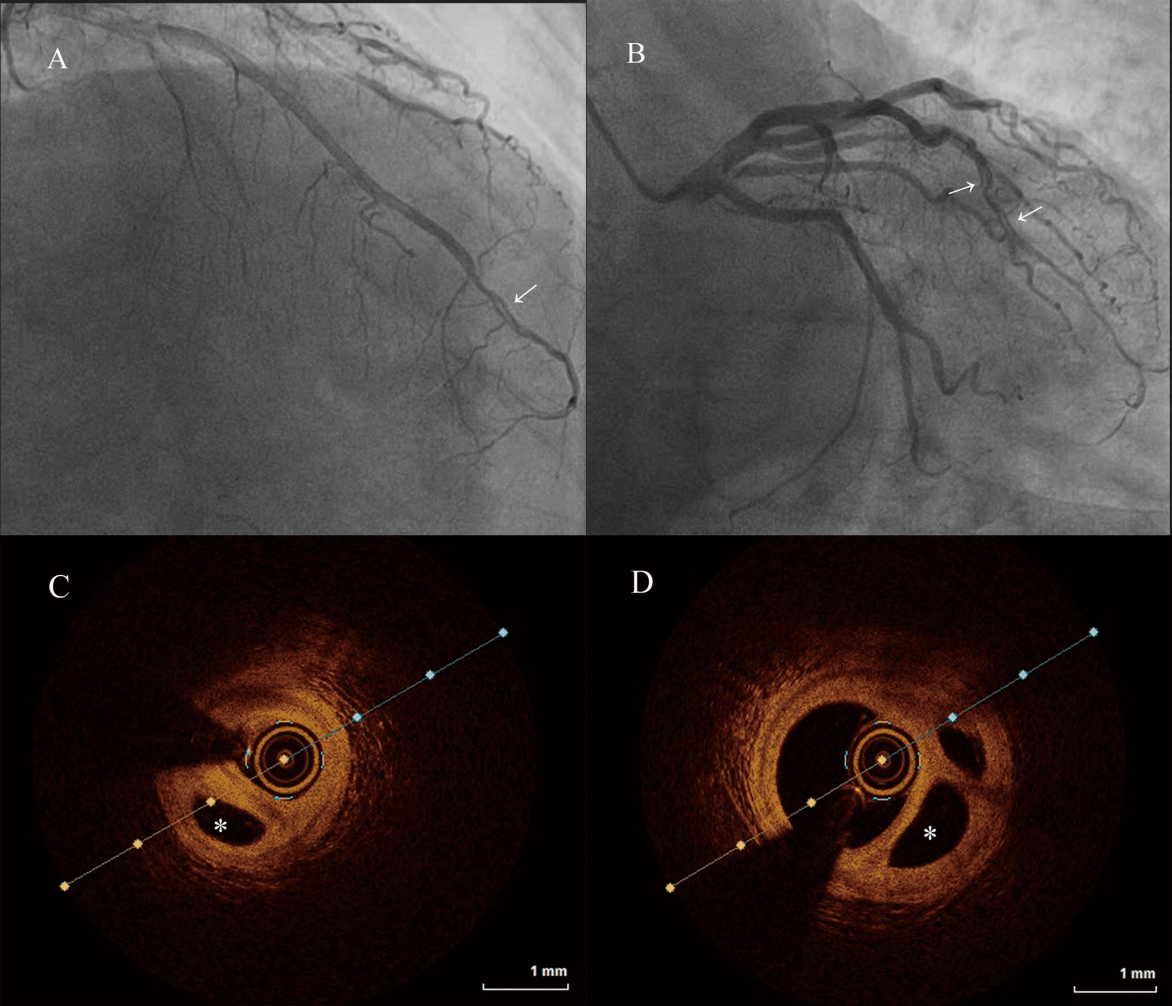
Conservative management of a woven coronary artery in the LAD A, B: coronary angiogram at baseline, highlighting the small, twisting channels (arrows); C, D: OCT images demonstrated multiple twisted channels without signs of thrombosis or dissection flaps, and extra lumina (asterisks) outside the vascular lumen were found on optical coherence tomography. OCT: optical coherence tomography; LAD: left anterior descending artery

A 63-year-old male was admitted to our cardiology ward on July 17, 2024, with a three-month history of intermittent chest pain. The patient had a 10-year history of hypertension and smoked for more than 30 years. Echocardiographic assessment revealed an ejection fraction of 63% with normal ventricular wall motion and no valvular involvement. Coronary angiography (CAG) disclosed a multiluminal structure in the distal segment of the LAD (Figures 2A, 2B). Subsequent OCT confirmed the presence of a woven coronary artery, characterized by cavities of varying sizes within the distal LAD and preserved vascular structure in all lumina (Figures 2C, 2D). Notably, there were no evident atherosclerotic plaques or thrombosis. Given the distal location of the lesion within the coronary artery, a conservative approach with OMT was recommended. The patient remained asymptomatic throughout a four-month follow-up period.

Case 3

**Figure 3:**
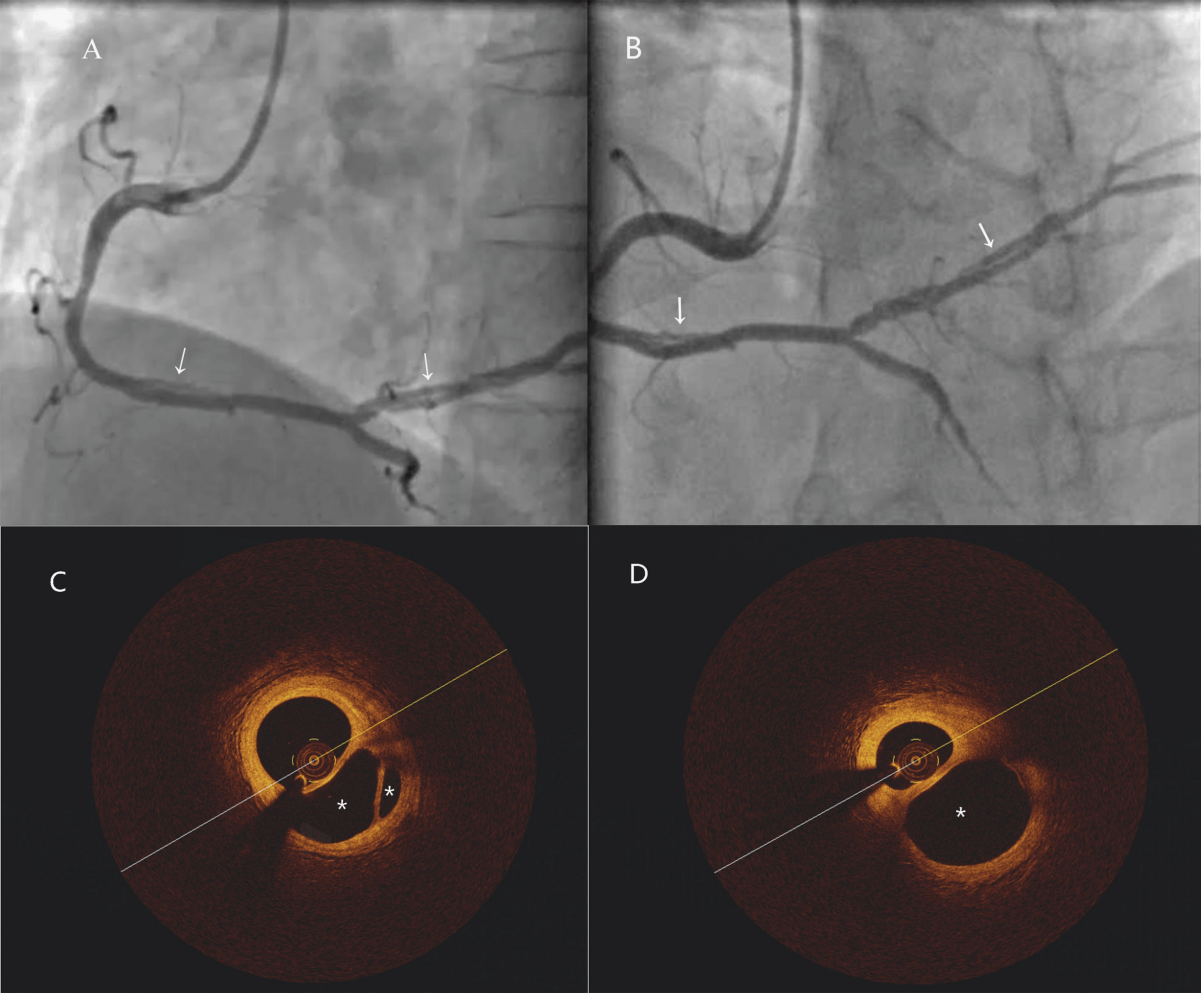
Conservative management of a woven coronary artery in the RCA A, B: coronary angiogram (CAG) showed tortuous and braid-like changes in the distal segments of the RCA and PLA (arrows); C, D: OCT images of the RCA revealed the woven structure with honeycomb cross-sections in each segment (asterisks), with each independent lumen maintaining an intact three-layer arterial wall structure. CAG: coronary angiography; OCT: optical coherence tomography; PLA: posterior lateral artery; RCA: right coronary artery

A 44-year-old male was admitted to our hospital with epigastric pain. The patient had been smoking for over 20 years and had no history of hypertension, diabetes, or hyperlipidemia. An electrocardiogram (ECG) revealed ST segment depression in leads I, augmented voltage left arm lead (AVL), and V4-V6, leading to a diagnosis of non-ST segment elevation myocardial infarction. CAG demonstrated a double-lumen structure with severe stenosis in the distal segments of the RCA and posterior lateral artery (PLA) (Figures 3A, 3B). OCT in the RCA revealed separate guidewires in each lumen and preserved vascular structure in all lumina (Figures 3C, 3D). The imaging showed multiple thin and tortuous epicardial arterial conduits that reconverged into a single lumen in the distal segments of the RCA and PLA. Notably, no thrombus or dissection flaps were observed. Fractional flow reserve (FFR) measurements were 0.78, and OMT was recommended. After a five-year follow-up, the patient remained free of any ischemic events.

## Discussion

In 1988, Sane reported the inaugural case of a 55-year-old female presenting with rheumatic heart disease and valvular disorders, marking the first documentation of WCA [3]. A case series published in 2020 challenged the accuracy of woven anomaly diagnoses, suggesting that some cases may be misattributed, potentially representing recanalized organized thrombi. It emphasized that coronary angiography alone was insufficient for diagnosis, as both pathological states can present as "braid-like lesions" [4].

OCT and intravascular ultrasound (IVUS) are advanced intravascular imaging techniques that facilitate a conclusive diagnosis through high-resolution visualization of the vessel lumen and the three distinct layers of the vessel wall [5-7]. OCT can support the congenital origin theory of WCA by revealing intertwined thin segments separated by fibrous tissue, intact arterial wall integrity without dissection, and high signal intensity with low signal attenuation [6,8-10]. Furthermore, the presence or absence of cross-communication is key to distinguishing WCA from recanalized thrombus. OCT reveals the recanalized thrombus as a "lotus-root" or "Swiss-cheese" appearance due to the presence of multiple interconnected channels within the thrombus, in contrast to the congenital woven anomaly, which lacks such cross-communication [11-13]. Moreover, OCT attributes that define WCA include a relatively intact three-layer vascular structure within each channel and a notable absence of significant coronary atherosclerotic disease [1,14]. This is in stark contrast to recanalized thrombus, which, while presenting a similar "honeycomb" appearance, is typified by a diffuse and heterogeneous fibrous or lipidic plaque. The central lumen in such thrombi is encircled by multiple channels with smooth borders that are interconnected, a feature crucial for differentiating it from WCA [15].

In our study, we identified that the woven-like alterations detected via coronary angiography are either congenital or acquired in nature. These alterations are typified by the presence of multiple thin vessels that share a common tunica media in the proximal segment and merge into a single lumen in the distal segment. The identification of three distinct layers within the vessel wall is indicative of a congenital etiology, while the detection of a pre-existing recanalized thrombus or evidence of cardiac dissection may indicate an acquired variant of the anomaly. Diagnosing WCA anomalies presents a formidable challenge, which intensifies when devising the most efficacious therapeutic approach. The dearth of literature on the long-term evolution of these coronary lesions exacerbates this complexity. Typically, WCA anomalies are benign and do not obstruct coronary flow. However, in rare instances, they may threaten coronary perfusion, triggering thrombogenesis. In such cases, a physiological evaluation is crucial for discerning the malignant variant. The therapeutic strategy for WCA patients is contingent upon the nuances of each clinical scenario, and there is a conspicuous absence of consensus or standardized guidelines. Treatment options span from conservative management to percutaneous interventions and bypass surgery. Patients who demonstrate WCA should be monitored, particularly in the absence of ischemic signs, and additional physiological assessment is needed. Yet, upon the manifestation of symptoms like angina, patients require additional diagnostic procedures to ascertain ischemia, which then informs decisions regarding interventions such as percutaneous or surgical revascularization [12,13,16]. FFR emerges as an invaluable tool in intervention decision-making [17]. FFR assesses coronary blood flow sufficiency by measuring the ratio of distal coronary pressure to aortic pressure in a stenosed vessel. A study harnessed fluid dynamics to explain the factors influencing pressure drop across a woven coronary artery, revealing that the number of channels and the length of the diseased segment are inversely related to arterial pressure. An increase in the length and number of channels correlates with a greater pressure drop, thereby reinforcing the notion that FFR is more effective in determining the adequacy of coronary blood flow compared to coronary angiography [18,19].

## Conclusions

In the context of coronary angiography, WCA manifests as a filling defect, often mimicking spontaneous coronary dissection or recanalized thrombus. Characteristic imaging patterns, including "honeycomb," "spiral," "braid-like," and "figure 8" configurations, are indicative of WCA. OCT emerges as an indispensable diagnostic adjunct, providing confirmatory evidence and mitigating the risk of procedural complications associated with misdiagnosis and facilitating the selection of therapeutic strategies.
